# Peptides Derived from Growth Factors to Treat Alzheimer’s Disease

**DOI:** 10.3390/ijms22116071

**Published:** 2021-06-04

**Authors:** Suzanne Gascon, Jessica Jann, Chloé Langlois-Blais, Mélanie Plourde, Christine Lavoie, Nathalie Faucheux

**Affiliations:** 1 Laboratory of Cell-Biomaterial Biohybrid Systems, Department of Chemical and Biotechnological Engineering, 2500 Boulevard Université, Université de Sherbrooke, Sherbrooke, QC J1K 2R1, Canada; Suzanne.Gascon@USherbrooke.ca (S.G.); Jessica.Jann@usherbrooke.ca (J.J.); 2 Département de Pharmacologie-Physiologie, Faculté de Médecine et des Sciences de la Santé, Université de Sherbrooke, Sherbrooke, QC J1H 5N4, Canada; Chloe.Langlois.Blais@USherbrooke.ca; 3 Centre de Recherche sur le Vieillissement, Centre Intégré Universitaire de Santé et Services Sociaux de l’Estrie–Centre Hospitalier Universitaire de Sherbrooke, Sherbrooke, QC J1G 1B1, Canada; Melanie.Plourde2@USherbrooke.ca; 4 Département de Médecine, Faculté de Médecine et des Sciences de la Santé, Université de Sherbrooke, Sherbrooke, QC J1H 5N4, Canada; 5 Institut de Pharmacologie de Sherbrooke, 3001 12th Avenue, N., Sherbrooke, QC J1H 5N4, Canada

**Keywords:** neurotrophin, bone morphogenetic proteins, MAPK, PI3K/AKT, cholinergic neurons, amyloid-β peptide, tau protein, metabolic pathway

## Abstract

Alzheimer’s disease (AD) is a devastating neurodegenerative disease characterized by progressive neuron losses in memory-related brain structures. The classical features of AD are a dysregulation of the cholinergic system, the accumulation of amyloid plaques, and neurofibrillary tangles. Unfortunately, current treatments are unable to cure or even delay the progression of the disease. Therefore, new therapeutic strategies have emerged, such as the exogenous administration of neurotrophic factors (e.g., NGF and BDNF) that are deficient or dysregulated in AD. However, their low capacity to cross the blood–brain barrier and their exorbitant cost currently limit their use. To overcome these limitations, short peptides mimicking the binding receptor sites of these growth factors have been developed. Such peptides can target selective signaling pathways involved in neuron survival, differentiation, and/or maintenance. This review focuses on growth factors and their derived peptides as potential treatment for AD. It describes (1) the physiological functions of growth factors in the brain, their neuronal signaling pathways, and alteration in AD; (2) the strategies to develop peptides derived from growth factor and their capacity to mimic the role of native proteins; and (3) new advancements and potential in using these molecules as therapeutic treatments for AD, as well as their limitations.

## 1. Introduction

Alzheimer’s disease (AD) is a neurodegenerative disorder characterized by a progressive decline of cognitive and behavioral functions, with typical symptoms such as memory loss and language or problem-solving difficulties [1]. AD is the leading cause of dementia, accounting for 60 to 80% of cases, and currently affects more than 50 million people worldwide [1]. Moreover, the World Health Organization (WHO) estimates that 131 million people will suffer from AD by 2050 [2], ensuring a global public health priority.

The etiology of AD is associated with changes in synaptic signaling, loss of synapses, and neuron degeneration [3]. It has long been reported that the dysregulation of the cholinergic system in the basal forebrain, a master regulator of executive and mnemonic functions, is linked to memory loss/cognitive decline in AD [4,5]. The cortical cholinergic denervation remains one of the earliest, most severe, and most consistent transmitter changes observed during AD progression, which led to the formulation of the “cholinergic hypothesis” [6].

The histological hallmarks of AD are the accumulation of dense extracellular deposits, also known as senile/amyloid plaques, and intracellular neurofibrillary tangles in several brain regions such as the basal forebrain, frontal lobe, hippocampus, cingulate gyrus, amygdala, substantia nigra, several brainstem nuclei, and the cerebral cortex [7]. The amyloid plaques are caused by the accumulation and aggregation of amyloid-β (Aβ) peptides (mainly Aβ_1–42_ but also Aβ_1–40_ peptides) generated by the consecutive cleavage of the amyloid-β precursor protein (APP) by β- and γ-secretases [8]. The neurofibrillary tangles are formed when the neuronal microtubule-associated protein tau is abnormally hyperphosphorylated by kinases such as glycogen synthase kinase 3β (GSK3β), leading to its release from the microtubule and intracellular aggregation into bundles of filaments [9]. It causes neuronal dysfunctions such as axon integrity and vesicular transport impairment [9,10].

The “amyloid hypothesis” (also known as the amyloid cascade hypothesis) has been the mainstream explanation for the pathogenesis of AD for over 25 years, but is still a highly controversial topic in the field. This hypothesis suggests that the accumulation and deposition of Aβ peptides is the initiating factor that triggers a cascade of disease-causing processes such as tau-tangle formation, neuroinflammation, synapse dysfunction, and cell death, which ultimately leads to dementia [11]. Despite ongoing debates about this hypothesis, evidence supports the idea that an imbalance between production and clearance of Aβ peptides is the initiating event of AD pathogenic processes [12]. The strongest evidence is that all the dominant mutations causing the familial (early onset, Mendelian-inheritance) form of AD reside either in APP or presenilin (catalytic subunit of γ-secretase), and result in increased production of Aβ_1–42_ or self-aggregation propensity of resultant Aβ peptides [11]. The overexpression of APP due to duplication of chromosome 21 in trisomy 21 (Down’s syndrome) has also been reported to cause an early appearance of Aβ_1–42_ plaques and development of AD at an early age (about 50% of people with Down syndrome who are in their 60s have AD) [1]. Furthermore, the amyloid hypothesis is also strongly supported by the identification of protective mutation of APP that results in lifelong decrease in APP cleavage into Aβ and reduced risk of AD [13]. While these genetic modifications greatly increase the AD risk, they are rare (1–6% of AD cases) [14]. Indeed, more than 95% of AD cases belong to the sporadic (late onset) form of AD (LOAD), which is caused by complex genetic and environmental factors. Apolipoprotein E4 (APOE4) is the most prevalent and important genetic risk factor for LOAD [15], with an estimated 3 to 12 times increased risk of LOAD [1]. APOE4 has been reported to have both amyloid-related and amyloid-independent effects, including reduced Aβ clearance by the blood–brain barrier (BBB) and decreased Aβ plaque load, tau tangle formation, and regulation of microglia linked to the triggering receptor expressed on myeloid cells 2 (TREM2) (also linked with high risk of LOAD [16]), proinflammatory activation, impaired glucose and lipid metabolism, and compromised vascular homeostasis [17,18,19]. Furthermore, several genome-wide association (GWAS) studies identified multiple AD-risk genes that could be linked with the Aβ cascade and/or tau pathology, but also to cholesterol and lipid metabolism, immune system and inflammatory response, and vesicle trafficking [20,21,22]. These reports highlight the complexity and multifactorial nature of AD.

## 2. Current Strategies Targeting AD Development

Current treatments of AD only alleviate symptoms for a short period, and there is still no cure for this disease or a way to stop or delay its progression. Presently, the only Food and Drug Administration (FDA)-approved treatments for AD are primarily acetylcholinesterase inhibitors (donepezil, rivastigmine, and galantamine), targeting the cholinergic system dysfunction [1,23] and memantine, an antagonist of the *N*-methyl-D-aspartate receptor (NMDAR) involved in chronic excitotoxicity and synaptic dysfunction [1,24]. However, these treatments have only modest and transient effects, and do not stop the progression of the disease [25,26].

In recent decades, many therapeutic approaches targeted the amyloid cascade components. Consequently, many clinical trials have been directed toward Aβ-lowering strategies, including interference with the amyloidogenic processing of APP, mainly with β- and γ-secretase inhibitors, and removing Aβ oligomers and plaques with monoclonal antibodies [22,27]. Unfortunately, until now, no therapy directed at reducing Aβ has been successful, resulting in either no cognitive benefit, or even worsening cognitive outcome or inducing major side effects [28,29,30,31]. However, a recent phase 2 trial of Donanemab, a humanized IgG1 antibody that targets a modified form of Aβ present only in established plaques, showed modest inhibition of cognitive and functional decline in early symptomatic AD patients [32]. While encouraging, longer and larger trials are necessary to study the efficacy and safety of Donanemab in AD. Therapeutic strategies targeting tau are also under investigation, including inhibitors of tau kinases and tau aggregation, and immunotherapy [27,33]. As for Aβ-target therapies, none of the tau-targeted therapies have been successful yet, and the only treatment that has reached a phase III trial is the tau aggregation inhibitor TRx0237 (LMTX™) [33].

Given that therapeutics targeting the main components of the Aβ cascade hypothesis failed in the late stage of clinical trials, these strategies have been reconsidered, and other strategies are being developed. Multiple promising targets to prevent AD or its progression have been identified [34]. The neuroinflammatory system, including astrocyte and microglia (key cellular regulators of neuroinflammation), and genetic variants linked to neuroinflammation, such as *TREM2*, have received great attention recently due to the notable correlation between the degree of neuroinflammation and the severity of AD [27,35,36]. Targeting, APOE; a major lipid transporter that plays a pivotal role in the development, maintenance, and repair of the central nervous system, and which polymorphism is a major risk factor for developing LOAD, is also in an early phase of therapeutic development [17,19,37]. Given that neurotrophin deficiency and dysregulation is closely associated with the pathogenesis of AD [38], supplementation of neurotrophic factors (nerve growth factor (NGF) and brain-derived neurotrophic factor (BDNF)) is currently a potential therapeutic approach to treat AD [27]. Using AD animal models, this treatment has proven its efficacy to ameliorate learning deficit [39,40]. A phase II clinical trial using adenoviral vector to deliver NGF (AAV2-NGF) to the basal forebrain (a region rich in cholinergic neurons) of AD patients demonstrated the feasibility of this approach [41]. The sections below describe the role of different growth factors in the central nervous system (CNS), their alteration in AD pathology, and potential uses as therapeutic treatments for AD.

## 3. Growth Factors in Brain Function and AD

### 3.1. Neurotrophins

The neurotrophin family includes NGF, BDNF, neurotrophin-3 (NT-3), and neurotrophin-4/5 (NT-4/5). Besides being essential during the development of the nervous system, they play a crucial role in the survival and phenotype maintenance and regeneration of specific types of neurons into adulthood [42]. In addition, they are implicated in the pathogenesis of certain neurodegenerative diseases, such as AD, and are thus targeted as potential therapeutic solutions for this disease.

#### 3.1.1. Structure

The human *NGF* gene is located on the proximal short arm of chromosome 1 (1p), while human *BDNF*, *NT-3*, and *NT-4/5* genes are located on chromosome 11 (11p), 12 (12p), and 19 (19q) respectively [43,44]. The *BDNF* gene has a very complex structure [45,46]. The human *BDNF* gene consists of nine functional promoters and one protein-coding 3′ exon that is spliced together with one of the nine noncoding 5′ exons or two noncoding exons unique to humans (Vh and VIIIh), leading to several mRNA splice variants [46]. The splice variants are expressed in response to specific stimuli [47]. For example, the translation of *Bdnf* transcripts containing exon-IV and -VI is directly or indirectly regulated by changes in neuronal activity, and may be linked to pathologies related to depression and memory disorders in the rat model [48,49,50]. The expression of specific *Bdnf* exons is regulated by epigenetic mechanisms in the adult rat brain during memory consolidation [51].

After synthesis in the endoplasmic reticulum, the precursor form of neurotrophin includes a signal sequence and a prodomain, followed by the mature protein sequence. The prodomain is cleaved intracellularly and/or extracellularly to release the mature protein. The cleavage of proNGF to obtain the mature form of NGF (mNGF) involves a CNS extracellular protease cascade leading to the activation of plasmin [52]. Both proNGF and mNGF are biologically active and can induce an antagonist effect on the maintenance of the cholinergic neuron phenotype [53]. Mature neurotrophin can also be degraded by enzymes such as matrix metalloprotease-9 (MMP-9) [52]. The process leading to the maturation of proNGF to mNGF, as well as the degradation of mNGF by MMP, is called the NGF metabolic pathway [54,55].

**Figure 1:**
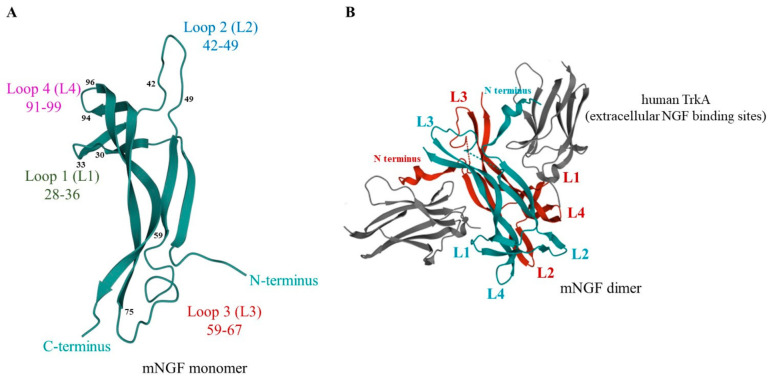
Structure of (**A**) mNGF (PDB ID: 1 BET) monomer [60]. The exposed β-turn loops L1 (residues 28-36), L2 (residues 42-49), L3 (residues 59-67) and L4 (residues 91-99) were used to design peptides. (**B**) The mNGF dimer (red and blue)-TrkA extracellular domain (black) binding sites (PDB ID: 2IFG [61]) [58,62].

The mature neurotrophins are evolutionarily conserved with a high sequence homology between vertebrates [43,56]. In addition, the mature NGF, BDNF, NT-3, and NT-4/-5 share 50% amino acid residue identity [57]. They also associate noncovalently into homodimers, with each monomer presenting a cysteine “knot” with the characteristic loop formation and a tertiary fold. These monomers (118 or 119 amino acids) are characterized by seven β-strands connected by three exposed β-turn loops (L1, L2, L4) and an additional loop L3 (Figure 1) [58,59]. All of these exposed sites may be accessible for interaction with neurotrophin receptors.

#### 3.1.2. Neurotrophin Receptors and Signal Transduction

The neurotrophin homodimers interact with two distinct classes of receptors: p75 neurotrophin receptor (p75NTR), which is a member of the tumor necrosis receptor superfamily, and tropomyosin receptor kinase (Trk) (Figure 1) [63]. Sortilin, a member of the Vps10p-domain family of transmembrane receptors, acts as a coreceptor of p75NTR [64].

The Trk family is composed of three Tyr kinase receptors: TrkA, TrkB, and TrkC. TrkA is expressed in the cortex and hippocampus, while TrkB and TrkC are expressed in both axonal and dendritic compartments in hippocampal, cortical, and cerebellar neurons [65].

The p75NTR receptor can interact with all neurotrophins in their pro- and mature forms [64]. TrkA mainly recognizes NGF, whereas BDNF and NT-4/5 interact with TrkB, and NT-3 binds TrkC; p75NTR regulates the specificity as well as affinity of Trk receptors for their neurotrophin ligands [66]. The affinity of NGF for both p75NTR and TrkA is quite similar (Kd around 1–2 nM) [64,67]. However, TrkA receptors co-expressed with p75NTR have a higher affinity for NGF (Kd 2.8 × 10^−12^ M) [67].

The extracellular domain of p75NTR consists of four cysteine-repeat domains, with two of them being implicated in the interaction with neurotrophins. The mNGF has two binding epitopes for p75NTR: the first one involves positively charged residues in L1 and L4 loops, whereas the second one involves hydrophilic residues from the highly conserved loop L3 and the C-terminus [68]. The p75NTR receptor also has single transmembrane and cytoplasmic domains, the latter containing a “death domain”.

The extracellular domain of TrkA contains three leucine-rich 24-residue motifs (LRR1-3) flanked by two cysteine clusters (CR). Two immunoglobulin-like C2-type domains (Ig-C2) are adjacent to these structures. Using the crystal structure of NGF/TrkA-d5 complex at 2.2 Å resolution, Wiesmann et al. found that the Ig-C2 domain (TrkA-d5) closest to the cell membrane is sufficient for the binding of mNGF through its L2 and L4 loops [68]. Each Trk receptor also has single transmembrane and cytoplasmic domains. The latter contains the tyrosine (Tyr) kinase activity region surrounded by phospho-Tyr residues involved in the recruitment of signaling and adaptor proteins of specific signaling cascades [63]. However, the tyrosine kinase domain is missing in some isoforms of TrkB and TrkC [69].

**Figure 2:**
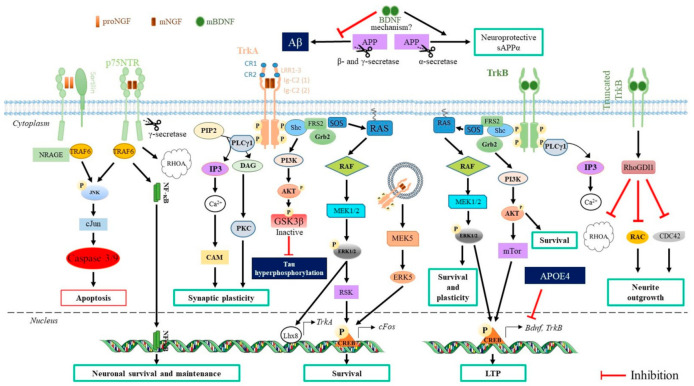
The NGF and BDNF signaling pathways and their roles in healthy and AD brains [70,71,72,73,74,75]. CAM: calmodulin kinase; DAG: diacylglycerol; mBDNF: mature form of BDNF (monomer); mNGF: mature form of NGF (monomer); RSK: ribosomal S6 kinase; TRAF: TNFR-associated factors. The figure was created using Servier Medical Art (https://smart.servier.com; 30 April 2021).

Upon binding, NGF and BDNF induce the dimerization of their cognate Trk full-length (Trk-FL) receptors. The cytoplasmic kinase domain of Trk receptors is in turn activated, and an autophosphorylation of their tyrosine residues occurs. These phosphorylations trigger the specific recruitment of adaptor proteins, the proto-oncogene tyrosine-protein kinase Src homology 2 domain containing (Shc), the fibroblast growth factor receptor substrate 2 (FRS2), and the phospholipase Cγ (PLCγ) (Figure 2).

Following this recruitment, Shc is Tyr-phosphorylated and stimulates the mitogen-activated protein kinase (MAPK)/extracellular signal-regulated kinase (ERK1/2) and phosphoinositide 3-kinase/protein kinase B (PI3K/AKT) pathways [76]. The Ras/MAPK/ERK1/2 pathway induces the activation of the transcription factor cAMP response element-binding protein (CREB), which is critical for early-response gene expression (e.g., c-Fos). Furthermore, NGF, through the MAPK/ERK1/2 pathway, potentiates *TrkA* transcription by the homeobox transcription factor LIM homeobox 8 (Lhx8) [77].

The adaptor protein PLCγ cleaves phospholipids to generate two second messengers, inositol 1,4,5-trisphosphate (IP3) and diacylglycerol (DAG), respectively leading to intracellular release of Ca^2+^/calmodulin kinase (CAM) activation and protein kinase C (PKC)-mediated signaling. The PLCγ1/Ca^2+^CAM/CREB pathway is involved in synaptic plasticity [78].

A transactivation of the Trk-FL receptors can also be initiated by G-protein-coupled receptors (GPCRs) such as adenosine A2A receptors [79]. Such transactivation can favor a neuroprotective effect [80].

While commonly considered dominant-negative receptor isoforms unable to signal, truncated TrkB, such as TrkB.t1 (which has a truncated C-terminal domain), inhibits Rho GTPase signaling by interacting with the Rho GDP dissociation inhibitor (RhoGDI1) [81,82,83]. This inhibition induces cytoskeletal rearrangement in neuronal cells [82]. However, the truncated Trk signaling and function in normal brain and neuropathologic conditions are complex and still under investigation (for a review, see [84]).

The p75NTR receptor has no intrinsic catalytic activity, but upon mature neurotrophin binding, its cytoplasmic domain can interact with adaptor proteins to activate downstream signaling molecules, including nuclear factor kB (NF-kB). Binding of pro-neurotrophin to p75NTR activates the JNK-caspase-3 mediated pathway, NF-κB pathway, and RhoA pathway [85,86]. However, the BDNF Val66Met polymorphism alters its prodomain structure, inducing different bioactivity due to impaired interaction with the sortilin receptor [87].

Both proBDNF and the mature form of BDNF (mBDNF) can also have an antagonist effect by binding with high affinity to p75NTR-sortilin and TrkB, respectively [88]: proBDNF/p75NTR-sortilin induces neuronal apoptosis [89], whereas mBDNF/TrkB protect the hippocampal neurons from glutamate-induced cell death [90,91]. An imbalance in the proBDNF:mBDNF ratio may therefore be involved in neuronal degeneration.

#### 3.1.3. Effects of Neurotrophins on the CNS Cells

- NGF

While BDNF mRNA in the adult human brain is found in the hippocampus, cerebral cortex, hypothalamus, and cerebellum, NGF mRNA is mainly expressed in the cortex and hippocampus [46,92,93]. The NGF-responsive neurons in the CNS are the cholinergic neurons of the basal forebrain (BFCNs) and striatum. The BFCNs, which possess extended axons throughout the hippocampus and neocortex, play a crucial role in learning and memory functions [94,95]. NGF, after its release by the postsynaptic cortical and hippocampal neurons, binds to TrkA and is retrogradely transported along the axon to the BFCN bodies. It can then initiate signaling cascades, leading to the maintenance of BDNF phenotype in adult CNS [55,96]. Indeed, NGF/TrkA signaling ensures the activation of genes encoding for cholinergic differentiation markers such as acetylcholine synthesis enzyme (ChAT) and the vesicular acetylcholine transporter (VAChT) [97]. Nevertheless, while the hippocampus of P20–P25 homozygous TrkA knockout mice (TrkA^−/−^) presents a great deficit in cholinergic fiber density, the cholinergic innervation of 28-day-old NGF knockout mice is not altered [98,99]. Importantly, Eu et al. recently reported a decrease in cholinergic fiber density in the hippocampus, but not in the cortex of 12-week-old *Ngf* gene knockout mice [100] (Table 1). An atrophy and loss of septal cholinergic neurons with deficits in memory and learning were also observed in heterozygous mutant mice (NGF^+/–^), which showed a decreased level of both NGF mRNA and protein [101]. In addition, chronic inhibition of the maturation of proNGF (but not proBDNF) with α_2_-antiplasmin treatment in the medial prefrontal cortex of normal adult rats led to a local loss and atrophy of cholinergic terminals paralleled by cognitive impairment. Interestingly, the number of dopaminergic, noradrenergic, glutamatergic, and GABAergic boutons were not affected. This cholinergic degeneration prevents the consolidation and retrieval of a new memory in rats [102].

- BDNF

BDNF has an important role in synaptic plasticity, including long-term potentiation (LTP) in the hippocampus of the adult brain, and is therefore involved in learning and memory consolidation [113,114]. BDNF is produced in the entorhinal cortex and then undergoes anterograde transport to the hippocampus [115]. mRNA expression encoding BDNF is increased in the hippocampus of rats that acquired spatial memory [116]. Moreover, using a hidden-platform water-maze task, Gorski et al. found that forebrain-restricted BDNF mutant mice (Emx-BDNFKO) present profound impairments in hippocampus-dependent learning [117]. Furthermore, BDNF can regulate the expression of two ionotropic glutamate receptors important for LTP: the alpha-amino-3-hydroxy-5-methyl-4-isoxazolepropionate (AMPA) and NMDAR [118].

Importantly, recent studies showed that the ratio of proBDNF to mBDNF is important for the synaptic plasticity. APOE4 epigenetically prevents *BDNF* transcription through the nuclear translocation of histone deacetylases 4 and 6 in human neurons [119]. It can also block the secretion and conversion of proBDNF to mBDNF [120]. Unlike mBDNF, proBDNF decreases the strength of the synapses [54,121].

#### 3.1.4. Effects of Neurotrophins on AD Hallmarks

- NGF

NGF mRNA levels are not decreased in the cerebral cortex of patients suffering from AD [122,123]. In contrast, using regional hippocampal dissections, Ginsberg et al. observed a downregulation of mRNAs for NGF and TrkA in patients suffering from mild cognitive impairment or AD compared to healthy subjects [124].

Recent studies highlight the role played by the NGF metabolic pathway, which is strongly affected in AD, leading to an imbalance in the proNGF:mNGF ratio [54,125]. The degradation of mNGF is promoted in AD due to an increase in MMP-9 activity, while the proNGF level is enhanced [126,127,128,129]. Indeed, an inhibition of the plasminogen activator factor in AD brain prevents the cleavage of proNGF to NGF by extracellular plasmin [130].

Unlike TrkA, the expression of p75NTR is not altered in BFCNs during the progression of the dementia [131]. An increase in the proNGF:mNGF ratio has been shown to be sufficient to alter the phenotype of BFCNs, inducing a downregulation of TrkA and ChAT protein expression, as well as degenerative retrograde alterations at their somatodendritic level [53]. Furthermore, proNGF extracted from AD frontal cortex can induce apoptosis in 3T3 cells expressing human p75NTR, while no effect was induced by proNGF isolated from a comparably aged control brain. This apoptosis depends on the γ secretase shedding of p75NTR [132]. The activation of PI3K/AKT and MEK/ERK pathways downstream of Trk was shown to prevent the apoptosis induced by proNGF [133].

Furthermore, NGF binding to TrkA has been suggested to promote the amyloidogenic cleavage of APP. A loss of the NGF/TrkA signaling could be linked to amyloid peptide deposition and tau abnormalities [134].

The use of exogenous mature NGF to restore the cholinergic system and treat AD hallmarks has therefore drawn considerable attention (for a review, see [5,55,135]). However, the delivery of NGF to brain neurons via peripheral vein administration is limited due to its molecular weight (despite its 13 kDa) and polarity that limit its transport across the BBB [136,137]. Furthermore, NGF had a plasma half-life of 7.2 min (normal adult rat) [136], and its intravenous administration in healthy human subjects can initiate diffuse myalgias in neck and throat muscles [138].

Intraventricular NGF administrations were therefore used to bypass the BBB and directly target the brain. For example, Hefti et al. performed repeated intraventricular injection of NGF (10 µg, twice weekly for 4 weeks) in adult rats with partial lesions of the cholinergic septo-hippocampal pathway. They observed a significant increase in hippocampal ChAT activity on the lesioned sides treated by NGF in comparison to the untreated ones [139]. Moreover, mouse NGF or recombinant human NGF (rhNGF, 625 µg per intraventricular injection for a total of eight injections) in monkeys prevents the progressive degenerative changes that occur in BFCNs following transection of their axons in the fornix [140,141]. A limited clinical trial of intracerebroventricular NGF administration (up to 3 months) on three patients suffering from AD did not demonstrate clear cognitive amelioration, although a few neuropsychology tests showed slight improvements. Unfortunately, several negative side effects, such as back pain and weight loss, were also reported [142].

The success of NGF approach strongly depends on the spatial and temporal delivery of the neurotrophins that must be controlled to avoid any side effect as described above [55]. The efficiency of other routes of NGF administration, such as intraocular or intranasal delivery, are still under investigation [143,144]. Other approaches based on *NGF* gene therapy include stereotactic surgery [145] or cell therapy [146]. Rafii et al. showed that bilateral stereotactic administration of adeno-associated virus serotype 2 delivering NGF (AAV2-NGF) to the nucleus basalis of Meynert can induce the synthesis of biologically active NGF without adverse events [145]. Nevertheless, no conclusion on cognitive outcomes arises from this study due to the small number of participants and lack of prospective control subjects [145]. A phase II clinical trial that included 49 AD patients recently confirmed that AAV2-NGF delivery was well-tolerated over 2 years, but no clinical cognitive outcomes were observed compared to the control group [41]. The use of transplanted cells (NGF cell therapy) also requires more studies on the inflammatory responses induced in the brain [147].

Thus, there are important challenges remaining in using NGF treatment, but there is still enthusiasm regarding this strategy for treating AD patients.

- BDNF

Several studies have shown that *BDNF* gene expression, as well as proBDNF and mBDNF levels, are decreased in the cortex, hippocampus, and basal forebrain in AD-affected brains [148,149,150,151] (Table 1). The decrease in BDNF expression appears to correlate with the degree of cognitive deficits in humans [152]. TrkB mRNA levels are downregulated in patients suffering from both mild cognitive impairment and AD compared to healthy patients in both CA1 pyramidal neurons and regional hippocampal dissections [124]. TrkB downregulation also correlates with the abundance of neuritic plaques and neurofibrillary tangles [153]. Several BDNF-mediated functions are altered in AD by β-amyloid peptides, as well as tau pathology, through the glucocorticoid receptor pathway [154,155]. The BDNF signaling impairment induced by Aβ might involve NMDAR dysregulation [156]. Interestingly, Aβ selectively increases mRNA levels for the truncated TrkB, and induces the cleavage of TrkB by calpain [157]. The truncated TrkB:TrkB-FL ratio is increased in hippocampal and cortical postmortem samples from AD subjects [148,149].

Since altered BDNF/TrkB signaling has been involved in AD pathology, various therapeutic approaches, such as exogenous mature BDNF delivery, *BDNF* gene therapy, and cell therapy, have been investigated [158,159]. Interestingly, unlike NGF, BDNF can cross the BBB in a bidirectional manner [160] However, the BBB penetration of BDNF remains low [136]. Therefore, some BBB modulators, such as cadherin peptides (ADTC5), have been used to improve BDNF’s efficiency in crossing the BBB. Intravenous injection of BDNF with ADTC5 in transgenic APP.PS1 mice improved the cognitive performance of these AD mice compared to BDNF alone [103].

Some promising results were also observed using *Bdnf* gene therapy in aged nonhuman primates. The BDNF-treated monkeys showed a significant improvement in performance of their visuospatial discrimination tasks [159].

Braschi et al. also recently found that intranasal delivery of BDNF at 42 pmol can rescue memory performance of AD11 mice, a sporadic model of AD. Surprisingly, this treatment has no effect on Aβ burden, tau hyperphosphorylation, or cholinergic deficit, whereas it induces a drastic decrease of CD11b immunoreactive brain microglia [104]. However, the comparison with human is difficult, since aging human and the murine microglia signature strongly diverge [161]. For example, the proportion of morphologically activated microglia in postmortem human cortical tissue is correlated with the accumulation of pathologic characteristic of AD, such as the number of amyloid plaques and tau accumulation, worsening the cognitive decline [162]. However, such results open new perspectives on the use of BDNF to treat AD.

### 3.2. The Bone Morphogenetic Protein (BMP)

BMPs/growth differentiation factors (GDFs) belong to the transforming growth factor-β (TGF-β) superfamily (for a review, see [163]). Other members of this superfamily include the TGF-β (TGF-β 1-3), nodal, activins, glial-derived neurotrophic factor (GDNF) family, and anti-Müllerian hormone/Müllerian inhibiting substance. BMPs are classified into four subgroups in function of their sequence homology: (I) BMP-2/BMP-4 Drosophila decapentaplegic (dpp) subgroup (92% amino acid identities); (II) BMP-5/BMP-6/BMP-7/BMP-8 Drosophila 60A subgroup (less than 65% residue identities with BMP-2); (III) BMP-9/BMP-10 subgroup; (IV) BMP-12/BMP-13/BMP-14/) subgroup [164,165]. BMPs are well known for their involvement in bone formation and remodelling [163]. However, studies using knockout mice highlighted that BMPs have a crucial role in eye, kidney, brain, and heart development [166,167,168,169].

#### 3.2.1. Pro-BMP and Mature BMP Complexes

As already described for neurotrophins, pre-pro-BMPs contain a signal peptide (22 amino acid residues, pre-pro-BMP-9), a prodomain for folding and secretion (297 residues, BMP-9 prodomain), and a mature BMP domain (BMP) (110 residues, BMP-9) [170]. After signal-peptide removal, the pro-BMPs form dimers that are then cleaved by furin, favoring the formation of complexes by noncovalent association between the prodomain fragments and BMP [170]. After secretion, the pro-BMP/BMP complexes interact with extracellular matrix proteins to obtain a latent cross-armed conformation [171].

#### 3.2.2. BMP Receptors and Signal Transduction

**Figure 3:**
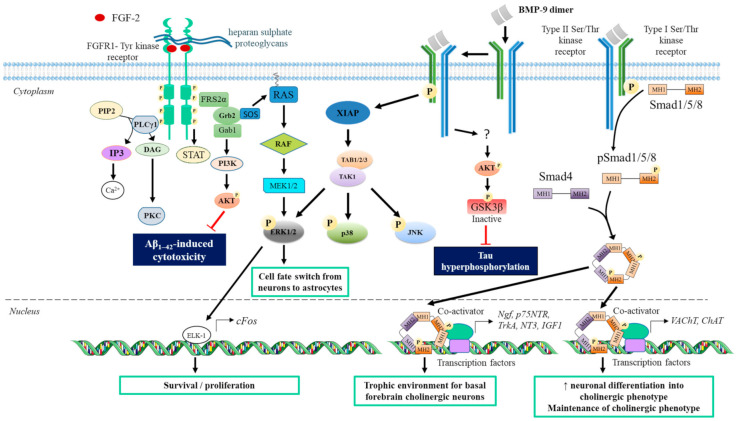
FGF-2 and BMP-9 signaling pathways and their roles in healthy and AD brains [106,185,188,189,190,191,192]. GAB1: Grb2-associated binder-1; SOS: salt overly sensitive; TAB1/2/3: TAK1 binding protein 1/2/3; TAK: transforming growth factor β-activated kinase 1; XIAP: X-linked inhibitor of apoptosis. The figure was created using Servier Medical Art (https://smart.servier.com; 30 April 2021).

BMP homodimer or heterodimer act on cells by binding to the heterotetrameric complex, comprising two dimers of Type I and Type II Ser/Thr kinase receptors (Figure 3) [172]. BMP dimers interact with Type I kinase receptors by their wrist epitopes, and Type II kinase receptors by their knuckle epitope [165,173,174]. These Type I or Type II receptors are characterized by a BMP-binding extracellular domain at their N-terminal extremity, a single pass transmembrane region, and a C-terminal intracellular domain containing the Ser/Thr kinase activity [175,176].

Members of the BMP family interact with three Type I (BMPR-1A or ALK3; BMPR-1B or ALK6; type 1A activin receptor ActR-1A or ALK2) and three Type II (BMPRII, ActRIIA and ActRIIB) Ser/Thr kinase receptors. Furthermore, BMP-9 can bind with a high affinity to another Type I Ser/Thr kinase receptor called ALK1 [165,173,177,178]. Most of Type I and Type II BMP receptors are present in the brain. BMPRII receptors are abundant in the cortex and hippocampus [179]. ALK-3 receptors are expressed in adult hippocampus-derived neural stem cells and astrocytes in the dentate gyrus and the hilar region, while ALK-6 expression is found in mature neurons [180]. BMP dimer binding to Type I and Type II Ser/Thr kinase receptors activates the canonical small mothers against decapentaplegic (Smad) 1/5/8 and/or MAPK signaling pathways [181,182,183]. Upon binding to BMP, the Type II receptors phosphorylate the Type I receptors at their GS motif. The activated Type I receptors phosphorylate, in turn, Smad 1, 5, or 8, which form a complex with Smad4. The Smad complexes are then translocated to the nucleus, where they interact with transcriptional coactivators to promote gene transcription such as *Id1-4* [180,184]. For example, BMP-9 induces the phosphorylation and nuclear translocation of Smad1/5/8 in SH-SY5Y cells [185]. The canonical Smad1/5/8 pathway is strongly regulated. Its activation can be prevented by several mechanisms, such as extracellular antagonists of BMP (Gremlin, Noggin, Chordin) [181], and the decreased surface availability of Type I and Type II kinase receptors due to their internalization through clathrin-dependent mechanisms [183]. The inactive membrane receptor BAMBI (decoy-receptor BMP and activin membrane-bound protein) can also block BMP signaling [186]. Other regulatory molecules of this signaling pathway act intracellularly. The pSmad1/5/8 can be deactivated via their dephosphorylation by phosphatases such as the protein phosphatase magnesium-dependent 1A (PPM1A). The canonical Smad pathway can also be inhibited by inhibitory Smad (I-Smad, Smad6/7) [187]. BMP2/4 can induce the upregulation of genes encoding for I-Smad in adult hippocampus-derived neural stem cells [180,187].

#### 3.2.3. Effect of BMP on CNS

Members of subgroup I (BMP-2/BMP-4) and II (BMP-5/BMP-6/BMP-7/BMP-8) are found in the hippocampus and/or cerebral cortex of adult brain [179,184,193,194,195,196]. For example, in adult rat brain, BMP-5 is widely expressed in neurons, astrocytes, ependymal cells, and meninges [196]; BMP-6 in astrocytes, ependymal cells, and oligodendrocytes [197]; whereas BMP-9 is detected in the spinal cord and septal area [198,199].

BMPs are involved in multiple events during the CNS formation and patterning (for a review, see [200]). However, even though several BMPs are widely expressed throughout the adult CNS, their role in its maintenance is still poorly understood [201]. Mira et al. found that BMP-2 dose-dependently decreases the percentage of proliferating adult hippocampus-derived neural stem cells cultured in medium supplemented with fibroblast growth factor 2 (FGF-2) [180]. BMP-4 also decreases the number of neural stem cells that enter the cell cycle. The deletion of both *Bmpr1a* and *Smad4* genes in these neural stem cells confirms that BMPR1a-Smad4 signaling is involved in cell quiescence induced by BMP-2/BMP-4 [180].

Among BMPs, BMP-9 has generated a great interest since it promotes BFCN differentiation and maintenance, and may also prevent cerebral ischemia–reperfusion injuries [188,189,190,202] (Table 1). BMP-9 favors the differentiation of mouse septal neurons into the cholinergic phenotype both in vitro and in vivo, and increases the production of the neurotransmitter acetylcholine [188,198]. BMP-9 can also induce an increase in mRNA levels of Idb1 and Idb3 transcriptional regulators, fibroblast growth factor receptor 3 (FGFR3), and BMPRIa within 48 h in dissociated septal cells from embryonic day 14 mice [188].

Furthermore, intracerebroventricular administration of BMP-9 (16 ng/µL (8 ng/h) over a 6-day period prevents the loss of cholinergic neurons after a septo-hippocampal transection in mice. It increases the expression of NGF and its receptors, p75NTR and TrkA in hippocampus [203].

#### 3.2.4. Effect of BMP on AD Hallmarks

Increases in *Bmp2*, *Bmp4*, and *Bmp6* transcript levels have been reported in hippocampi of aged mice. Both BMP-4 and BMP-6 protein levels are more abundant in the cortex of old mice [204]. An upregulation of BMP-6 mRNA levels was also observed in the hippocampus and cortex of patients with AD [105]. Crews et al. suggested that BMP-6 in AD may have deleterious effects on adult hippocampal neurogenesis due to its inhibitory effect on stem cell proliferation [105]. Aβ_1–42_-containing plaques appear to play a key role in BMP-6 upregulation in AD, increasing BMP-6 mRNA and protein expression in the neural progenitor cells (Table 1) [105]. In the same way, an increase in BMP-4 levels was correlated with reduced hippocampal cell proliferation in a mouse model of AD [205]. The involvement of BMPs in CNS and neurodegenerative disorders such as AD is still under investigation [200].

Several studies have shown that BMP-9 might be a promising molecule to treat AD [106]. Using a AD mouse model (APP.PS1), which exhibit cholinergic defects, high accumulation of amyloid plaques, and cognitive impairments, Burke et al. found that intracerebroventricular infusion of BMP-9 (4 ng/h) for 7 days can reduce Aβ42-positive plaques in the cortex and hippocampus [106]. This treatment also favors the establishment of a trophic environment for BFCNs in the hippocampus by upregulating the expression of NGF, its receptors (p75NTR, TrkA), NT-3, and insulin-like growth factor 1 (IGF-1) [106]. Wang et al. have recently confirmed that BMP-9 injected intranasally into APP.PS1 mice (50 ng/g/d for 30 days), reduces senile plaque accumulation and restore cognitive function [107]. In addition, GSK3β, one of the kinases involved in the hyperphosphorylation of tau, is inhibited by its phosphorylation at Ser9 in the brain of BMP-9-treated mice. Indeed, the level of hyperphosphorylated tau in neurons in the cortex and hippocampus is decreased in BMP-9-treated APP.PS1 mice [107].

Therefore, BMP-9, through its action on BFCN, might be a promising molecule to treat AD. In addition, its receptor ALK1 is not affected at the early stage of AD [206]. However, BMP-9 acts as a dimer of around 24 kDa that cannot easily cross the BBB, and the use of supraphysiological doses is not only very expensive, but more importantly, may induce side effects.

### 3.3. FGF and Other Growth Factors

#### 3.3.1. FGF

To date, there are 22 mammalian FGFs (for a review, see [207]). They are classified into seven subfamilies based on their sequence homology and phylogeny involving the canonical FGFs, endocrine FGFs, and intracellular FGFs (for a review, see [208]). In human, FGF-2 has five isoforms of different molecular weights (18 kDa, 21 kDa, 22.5 kDa, 24 kDa, and 34 kDa) resulting from alternative initiations of mRNA translation [209]. There are only three isoforms in mouse: 22 and 21 kDa high molecular weight (HMW) isoforms, and one 18 kDa low molecular weight (LMW) isoform [210].

FGFs have a core region of around 120–140 amino acids forming 12 antiparallel β-strands (β1–β12). A heparan sulphate proteoglycan binding site involves the β1–β2 loop and parts of the region spanning β10 and β12. The core region is flanked by amino and carboxyl termini [211].

- Receptors and signal transduction

The 18 secreted FGFs can bind to four Tyr kinase receptors (FGFR1–FGFR4). These receptors, which share 46% sequence identity, have three extracellular immunoglobulin-like domains (IgI, IgII, and IgIII), a single transmembrane domain, and a cytoplasmic Tyr kinase domain [212]. FGFRs are expressed in different areas of the brain. For example, FGFR1 is widely expressed in the hippocampus and in various parts of the cortex, while FGFR4 is mainly found in the medial habenular nucleus. Both FGFR1 and FGFR4 are primarily neuronal, whereas oligodendrocytes and astrocytes express FGFR2 and FGFR3 [213,214,215]. Upon binding of two FGF ligands and two heparan sulfate proteoglycans as cofactors, the FGFR receptors dimerize and autophosphorylate, allowing the intracellular recruitment of PLCγ1 and FRS2α (Figure 3). FRS2α activates the Ras/MAPK and PI3K/AKT pathways [208], while PLCγ1 activates the Ca^2+^/CAM and PKC pathways. The FGFR kinase domain also initiates the signal transducer of activators of transcription (STAT) pathways [208].

- Effect of FGF on CNS and AD hallmarks

FGFs, such as FGF-2, play an important role during brain development [216]. FGF-2 also controls the neurogenesis through its involvement in the differentiation of new neurons in the adult dentate gyrus [217]. It is mainly synthesized by astrocytes in the CNS, and by microglia and neurons in the CA2 region of the hippocampus [218]. FGF signaling, especially the MAPK pathway, is crucial in the cell-fate switch from neurons to astrocytes in the developing mouse cerebral cortex [191].

FGFs, such as FGF-2 and FGF-21, have been shown to be beneficial to treat AD hallmarks (Table 1) [112,219]. While no difference was detected in the levels of LMW FGF-2 between AD patients and age-matched healthy controls, the expression of HMW FGF-2 isoforms was drastically decreased in AD patients [219]. Both FGF-2 HMW and LMW isoforms can protect against Aβ_1–42_-induced cytotoxicity in astrocytes through the activation of the PI3K/AKT signaling pathway [111]. *FGF-2* gene delivery by stereotaxic hippocampal injection induces a decrease of Aβ through microglial activation in AD transgenic APP.PS1 mice. In addition, it can restore spatial learning, hippocampal CA1 LTP, and neurogenesis in APP.PS1 or J20 mice, two AD mice models [110].

FGF-2 can be administered systemically and cross the BBB to produce its effects. For example, in 10.5-month-old female APP23 mice, a model of amyloid pathology, FGF-2 injected subcutaneously at 20-µg/kg per day for 3 weeks increases the number of astrocytes and limits the expression of inflammatory mediators. It also reduces the generation of Aβ as well as the phosphorylation of tau, while it restores the spatial memory [219]. The intranasal administration of FGF-2/chitosan seems more effective to deliver the growth factor to the brain in comparison to intravenous injection. It also induces a better improvement of spatial memory in rat with learning impairment after co-injection of Aβ_25–35_ and ibotenic acid [220]. These data suggest that intranasal administration of FGF-2 could have potential application in AD.

#### 3.3.2. Other Growth Factors

Other growth factors, notably ciliary neurotrophic factor (CNTF) (for a review, see [221]) and GDNF, are also interesting molecules because of their role in the progression of dementia [153]. Insulin growth factors like IGF-1 have also been tested in the context of AD. Low plasma levels of IGF-1 have been previously associated with decreased cognitive performance [222,223]. However, a recent study reported that older males with high level of IGF-1 showed poor concurrent cognition. Furthermore, high levels of IGF-1 beyond a threshold in middle-aged males are associated with a decline in future cognitive function [224].

The benefit of IGF-1 administration in the context of AD is also still under debate. Some studies found that peripheral administration of IGF-1 (50 mg/kg/day) facilitates the clearance of Aβ (Table 2) [225,226]. In contrast, IGF-1 delivery (50 mg/kg/day for 1 month) in 11-month-old Tg2576 mice has no beneficial effect on amyloid plaque load or Aβ levels [227]. Furthermore, inhibition of IGF-1 signaling seems to decrease AD hallmarks [228]. The administration of a potent inducer of circulating IGF-1 levels (MK-677) also fails to delay AD progression in a randomized trial [229]. Further investigations are therefore required to better understand the role played by IGF-1 and its signaling in AD.

## 4. Peptides Derived from Growth Factors

**Figure 4:**
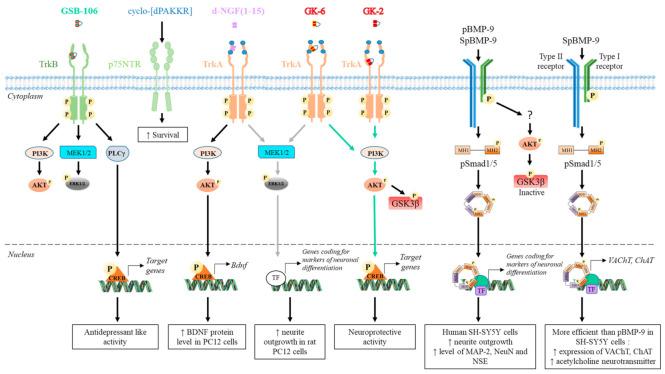
Signaling pathways activated by peptides derived from growth factors and their effect in vitro and/or in vivo [232,239,243,244,245]. The figure was created using Servier Medical Art (https://smart.servier.com; 30 April 2021).

To overcome the limitations encountered using native growth factors, alternative strategies involving biologically active small peptides have been developed (Table 2), in order to improve pharmacokinetic properties and BBB permeability, selectively activate targeted signaling pathways (biased signaling), and decrease the side effects compared to full-sized proteins [59,185]. Figure 4 summarizes the signaling pathways induced by the peptides and their subsequent effect on neuronal differentiation, cholinergic phenotype, and cell survival in vitro and/or in vivo.

### 4.1. Peptides Derived from Neurotrophins

#### 4.1.1. NGF

- Peptides derived from mNGF L1-L4 loops

At first, NGF-derived peptides were designed based on conserved amino acid sequences that have a high degree of hydrophilicity and may correspond to the receptor binding sites [246]. Longo et al. have therefore identified three sequences: ^26^TTATDIKGKEVTVLA (region C), ^64^VESGCRGIDSKHW (region A), and ^99^WRFIRIDTA (region B) [246], corresponding to potential active sites. Only the linear peptides derived from region C (C1 (ATDIKGKEVTV), C2 (DIKGKEVTV), and C5 (KGKE)) inhibited the neurite outgrowth induced by NGF in sensory neurons. These antagonist peptides cannot block the binding of NGF to its receptors [246]. However, Ibanez et al. found that the region C (^28^ATDIKGKEV^36^) contains two lysines (K32 and K34) that are involved in NGF binding to p75NTR [247].

The resolution of the structure of mNGF explains that the lack of secondary structure is why the linear peptides were unable to prevent NGF binding to the receptors. Indeed, X-ray crystallographic analyses of mouse NGF showed that exposed sequences are organized as three hydrophilic β-turn loops [248], later identified as L1, L2, and L4 loops, with an additional exposed L3 loop (Figure 1) [68]. The L1 and L4 loops are known to be involved in mNGF-receptor interaction [68]. Therefore, in addition to the region C corresponding to the L1 loop (^28^ATDIKGKEV^36^), three other sequences that are part of L2 (^42^VNINNSVF^49^), L3 (^59^RASNPVESG^67^), and L4 (^91^TTDEKQAAW^99^) were used to design linear or cyclic peptides [249]. Only the small cyclic peptides mimicking the three-dimensional β-turn conformation (C(30-35) CDIKGKEC; C(43-48) CNINNSVC; C(60-65) CASNPVEC; C(92-96) CTDEKQC) were very potent antagonists of NGF. More importantly C(92-96) can bind TrkA and inhibit the binding of NGF to the TrkA receptor [249,250].

Since the cyclization of the NGF-derived peptides mimicking β-turn loop structure gave promising results to develop an NGF mimetic, Longo et al. developed several peptides mainly based on the most efficient sequences ^29^TDIKGKEV^36^ and KGKE (C5) [251]. To obtain a stable oxidative peptide cyclization, penicillamine and cysteine were added at the N- and C- (amide) extremities of the peptide, respectively. Among the designed peptides, the cyclopeptide P7 (IPenKGKEVCT) has the greatest survival-promoting activity via p75NTR receptors. More importantly, only the dimerization of P7 allows a neurotrophic activity [251].

Other NGF dimeric mimetic peptides were developed based on the β-turn sequences of loops L1 and L4, which most significantly protrude outward and must play a major role in the interaction with the receptors [252]. Gudasheva et al. designed two dimeric dipeptides called GK-2 and GK-6 [230,231]. GK-2 is based on the L4 loop sequence ^93^DEKQ^96^. To stabilize its conformation and limit its degradation by peptidase, Asp93 and Gln96 are substituted by succinic acid residue and amide group, respectively. GK-6 is composed of the dipeptide fragment of the first loop Gly33–Lys34, protected at its N- and C-terminus, as described for GK-2 [231]. Both GK-2 and GK-6 were reported to mimic NGF activity through their capacity to activate TrkA receptors in HT22 neurons (Table 2) [231]. However, upon TrkA activation, GK-2 and GK-6 induce different signaling pathways (Figure 4) [243]. Like, NGF; GK-6 stimulates both the PI3K/AKT and MAPK/ERK1/2 pathways. In contrast, GK-2 only activates the PI3K/AKT pathway. GK-2 has a neuroprotective effect in several models of oxidative stress [230]. GK-6 also exerts a neuroprotective effect. Unlike GK-2, it induces the differentiation of rat PC12 pheochromocytoma cells, which requires the activation of the MAPK/ERK1/2 pathway (Table 2) [243]. Furthermore, in contrast to GK-6, GK-2 did not induce hyperalgesia, which is one of the primary adverse effects of NGF [243]. Therefore, even if both peptides are agonists of TrkA, they induce a biased signaling for a selective downstream pathway and different profiles of biological activity. These are promising examples of how the design of peptides based on different binding region of NGF is key in the development of pharmacological agents that target the desired neuronal activity of NGF without the main side effects.

GTS-115 (bis(N-gamma-hydroxybutyryl-L-lysyl-L-histidine)), a peptide derived from the β-turn sequence of loop L3, activates TrkA receptor and mediates its signal transduction through the MAPK/ERK1/2 and PI3K/AKT pathways. It also shows a neuroprotective activity on HT-22 cells cultured under an oxidative stress condition, but at a higher concentration range (10^–5^ to 10^–7^ М) compared with GK-2 [252].

Because of their reported neuroprotective effects, several of these peptides were also evaluated in vivo using animal models for various neurodegenerative diseases and ischemic stroke (Table 2).

- Linear peptides derived from the mNGF N-terminal region

Given that the amino acid residues 4 to 13 of mNGF (especially His-4, His-8, Ile-6, Phe-7, and Glu-11) play a crucial role in the interaction and activation of TrkA [253,254,255], they were used to develop peptides derived from the mNGF N-terminal (Table 2).

NGF(1–14) is a linear peptide that encompasses the first 14 amino acid residues of the human mNGF with the C-termini amidated to mimic the sequence within NGF [256,257]. It triggers a slower, but longer lasting, activation and phosphorylation of TrkA in comparison to NGF [257]. Interestingly, while NGF(1–14) is able to activate the PI3K/AKT pathway, leading to GSK3β inactivation and phosphorylation of the transcription factor CREB, it is unable to induce the phosphorylation of ERK1/2. As expected, since the MAPK/ERK pathway is inactive, NGF(1−14) does not favor the differentiation of PC12 cells [257]. However, these results were in contradiction with those published by Pandini et al., who observed an ERK1/2 activation in PC12 cells treated with NGF(1–14) [258]. The authors suggested that the number of cell passages may have influenced the cell response. It was also reported that the addition of Cu^2+^, known to accumulate in AD brain [259], alters the conformation of NGF(1–14) and significantly increases its proliferative effect [258].

NGF(1–14) is a monomer with no detectable propensity to dimerize [257]. Because dimeric peptides are more efficient to mimic native NGF, d-NGF(1–15), a dimeric form of NGF(1–14) peptide, was obtained via a cysteine-bridge linker of two monomeric units [232]. As shown for NGF(1–14), the secondary structure of d-NGF(1–15) is stabilized by Cu^2+^ ions; d-NGF(1–15) interacts with the d5 domain of TrkA and is a better TrkA activator than NGF(1–14). It induces the activation of the MAPK/ERK1/2 and PI3K/AKT pathways, triggers CREB phosphorylation, increases BDNF levels and secretion, and significantly increases neurite outgrowth in rat PC12 cells (Table 2 and Figure 4) [232]. Thus, use of this dimeric peptide is a promising strategy to restore the neurotrophin levels in neurodegenerative disease, such as AD.

- Peptides designed by combining sequences from mNGF loops L1 and L4 and the N-terminal region

In order to increase the efficacy, more complex NGF-mimicking peptides, such as NL1L4 and L1L4, were designed by combining sequences from well-known regions of mNGF-TrkA binding sites. The NL1L4 peptide incorporates sequence residues of the N-terminal region (His^4^-Asp^24^), and residues from the L1 and L4 loops (Thr^29^-Lys^34^ and Asp^92^-Gln^95^) that were cyclized to restrain their conformation flexibility and connected with a linker TGA [236]. The L1L4 peptide was designed as NL1L4, but without the N-terminal sequence. Both cyclic L1L4 and NL1L4 activate TrkA, but not TrkB. They also induce differentiation of DRGs and PC12 cells, whereas the linear form of these peptides did not, even at high concentration [236]. L1L4, which has the highest in vitro activity, is also effective in reducing neuropathic pain in a chronic sciatic constriction injury (CCI) model to an extent comparable to native NGF (Table 2) [236]. In addition, intrathecal administration of this peptide does not cause the algesic effect of NGF [236], and is thus a potential therapy for neuropathic pain and other brain disorders.

- Effect of NGF-derived peptides in the context of neurodegenerative diseases

Aβ has been reported to induce neuronal death by binding to p75NTR. Antagonistic peptides have been designed to prevent this adverse effect of Aβ. A cyclic peptide (CATDIKGAEC) was derived from mNGF-β hairpin loop L1 (residues 29-35), a region interacting with p75NTR, but in which KGE was replaced by KGA, a motif shared by NGF and Aβ [233]. This cyclic peptide inhibits NGF and Aβ_1–40_ binding to p75NTR, but not to TrkA, and prevents the neuronal death induced by Aβ_1–40_ in E17 rat cortical neurons (Table 2) [233].

Other NGF-derived peptides have shown a neuroprotective effect both in vitro and in vivo. The dimeric peptide GK-2 (described in the subsection “Peptides derived from mNGF L1-L4 loops”) had neuroprotective activities in several experimental models. It stimulated neuroprotective and neurogenesis activities in vivo in a rat model of ischemic stroke (Table 2) [235] and in several other experimental models of traumatic brain injury or degenerative diseases [59,260,261]. GK-2 counteracts the impaired cognitive functions in two AD rat models: (1) a surgical one (transection of the septo-hippocampal pathway), resulting in the development of cholinergic deficiency; and (2) a neurotoxic one (streptozotocin), reproducing the main pathological hallmarks (Aβ accumulation and tau phosphorylation) (Table 2) [234]. Importantly, GK-2 systemic administration, unlike the native NGF, did not cause hyperalgesia and weight loss in these in vivo experiments [243]. These results suggest that GK-2 may be a promising molecule to prevent the development of AD [234].

Through their capacity to target specific receptors and to selectively activate a subset of signaling pathways, NGF-derived peptides can favor neuroprotective and neurogeneration activities. These characteristics make these molecules very promising tools to fight neurodegenerative diseases. However, a lot of work remains to better understand the effect of such peptides on the complex AD pathogenesis.

#### 4.1.2. BDNF

- Linear peptides derived from mBDNF

Using neutralizing antibodies directed to identify active sites of mBDNF, five different linear tetrapeptides were designed (peptides B-1 to B-5). B-3, B-4, and B-5 peptides exert neurogenic and neurotrophic effects in mouse hippocampal neuronal cell culture. Both B-5 and B-3 were found to work as partial agonists and antagonists for TrkB activation, and also to induce the expression of TrkB and BDNF (Table 2) [237]. HNgfEE is another short peptide derived from the NGF sequence that shares similarities with the BDNF sequence. When conjugated to the surface of polymersome nanoparticles, this peptide can bind and activate the TrkB receptor in vitro [262]. However, to date the efficiency of these peptides have not been evaluated in vivo.

- Cyclic dimeric peptides derived from mBDNF loops L2 and L4

The cyclic peptides derived from BDNF were designed based on the X-ray crystallographic data obtained for mouse NGF and a BDNF/NT3 heterodimer [263], revealing the β-hairpin loop (L1-L4) regions in the BDNF primary sequence. In addition, specific site-directed mutagenesis and chimeric proteins (NGF with the L2 region of BDNF) showed that amino acid residues in the L2 loop are involved in the interaction with TrkB receptors [264,265]. Based on these information, four conformationally constrained cyclic peptides of various size and derived from the L2 loop were synthesized (L2-12, L2-10, L2-8, and L2-6). These peptides act as competitive antagonists of BDNF for TrkB. However, they have no survival-promoting activity [263].

Bicyclic dimeric peptides (disulfide-linked and amide-linked dimers) were then designed based on the L2-8 sequence (e.g., (H-CVCVSKGQLC-OH)_2_) in order to obtain potent peptides mimetic of BDNF. These peptides behave as partial agonists, promoting the survival (around 29% of the maximal survival effect of BDNF) of embryonic chick dorsal root ganglion sensory neurons. To improve the potency/efficacy of these compounds, tricyclic dimeric peptides (hybrids of the disulfide-linked and amide-linked dimers) were also designed to reduce conformational freedom and potentially favor the orientation of the two monomeric units for receptor dimerization. Although still partial agonists, these peptides were very potent in increasing neuronal survival (100- to 1000-fold more potent than the bicyclic disulfide-linked dimers) [266].

Other mimetic peptides of BDNF were designed based on the p75NTR-binding tripeptide motif KKR available on loop L4 of BDNF. For example, Fletcher et al. used a cyclic pentapeptide (cyclo-[dPAKKR]) that consisted of the KKR tripeptide constrained by a dPro-Ala linker. Unlike, BDNF; cyclo-[dPAKKR] cannot activate TrkB (Figure 4). However, it acts as a BDNF agonist favoring the survival of primary embryonic chick sensory neurons. Furthermore, this peptide is highly resistant to proteolytic degradation by plasma in vitro [244]. An alkyl amide-substituted analogue of this peptide (cyclo-[dPK(alkyl amide)KKR]), which may recruit the peptide to cellular membrane, was found to be over 60-fold more potent than cyclo-[dPAKKR] [267].

Based on these results, a dimer dipeptide named GSB-106 was derived from the BDNF loop L4 β-turn sequence D^93^SKK^96^, where Asp93 was replaced by a succinic acid residue, and Lys96 was replaced by an amide group [245]. Surprisingly, GSB-106 activates TrkB receptors as well as the downstream PI3K/AKT, and MAPK/ERK1/2 and PLCγ [268]. It was also recently reported that GSB-106 induces the phosphorylation of TrkB via a transactivation mechanism partially dependent on Src kinases in neuroblastoma SH-SY5Y cells. GSB-106 exerts a neuroprotective effect against glutamate toxicity on cells expressing TrkB. It also promotes survival of serum-deprived SH-SY5Y cells through TrkB/PI3K/AKT pathway activation, which inhibits apoptosis [268]. Therefore, GSB-106 mimics BDNF in its prosurvival activity.

- Peptides combining different regions of mBDNF

Long peptides derived from loops L3 and L4 in BDNF, Betrofin 3 (RGIDKRHWNSQ) and Betrofin 4 (SYVRALTMDSKKRIGWR), respectively, were synthesized as dendrimers composed of four monomers coupled to a lysine backbone. Both peptides can bind p75NTR and TrkB receptors and induce neurite outgrowth of primary cerebellar granule neurons [269]. Despite their effect on neuronal differentiation, the molecular weight of these dendrimers is high and may limit their delivery to the brain.

- Effect of BDNF-derived peptides in the context of brain trauma and diseases

There are few studies on the effect of the BDNF-derived peptides in the context of AD. However, their uses as antidepressants or to improve neurologic outcomes after brain trauma have been well documented [270,271]. For example, GSB-106 improves neurologic outcomes via PI3K/AKT and MAPK/ERK1/2 pathway activation in rat stroke model caused by transient middle cerebral artery occlusion [271]. Furthermore, GSB-106 administered intraperitoneally or orally exhibits antidepressant activity [270,272,273]. It also restores hippocampal neuroplasticity in a mice depression model induced by a chronic social defeat stress procedure (Table 2) [238]. GSB-106 has therefore successfully passed preclinical studies as a potential antidepressant.

### 4.2. Peptides Derived from BMP

#### 4.2.1. Peptides Derived from the Knuckle Epitope

Based on the previous work of Saito et al. on BMP-2 [274], our research team has designed two peptides, pBMP-9 and SpBMP-9, derived from the knuckle epitope of BMP-9 corresponding to the amino acid residues recognized by the Type II Ser/Thr kinase receptor BMPRII [275,276]. Like BMP-9, both peptides can activate the Smad canonical and PI3K/AKT pathways, and inhibit GSK3β, a well-known tau kinase (Figure 4) [239]. Both pBMP-9 and SpBMP-9 favor the differentiation of human neuroblastoma SH-SY5Y cells toward neurons better than BMP-9, with SpBMP-9 being the most effective to promote the cholinergic phenotype (Table 2) [239]. These results might be explained by a difference in pBMP-9 and SpBMP-9 affinity for BMPRII receptors.

Several studies have shown that BMP-9 can induce the synthesis of NGF both in vitro and in vivo, or act in synergy with other growth factors such as FGF-2 to promote the differentiation of cholinergic neurons [106,190,198]. We therefore verified whether SpBMP-9 can act with several growth factors (FGF-2, EGF, IGF-2) and neurotrophin NGF to promote cholinergic differentiation of SH-SY5Y cells [240]. Unlike its negative peptide NSpBMP-9, SpBMP-9 can potentiate the effect of both bFGF and NGF on SH-SY5Y cell differentiation toward the cholinergic phenotype. In contrast, there is no synergistic effect in terms of neurite outgrowth for cells stimulated with SpBMP-9 combined with IGF-2 or EGF [240]. These results showed that SpBMP-9 may be a promising molecule to treat AD by increasing the cholinergic phenotype in combination with other growth factors and inhibiting GSK3β. However, such small peptides must be protected to be delivered to the brain. SpBMP-9 was successfully encapsulated into composite nanoparticles made of alginate and chitosan for its intranasal delivery. SpBMP-9 released from the nanoparticles was still biologically active [277].

However, the evaluation of SpBMP-9′s efficiency to treat AD hallmarks first requires several in vivo studies using appropriate AD mice models.

#### 4.2.2. Peptide Derived from the Wrist Epitope

GBMP1a was designed based on the BMP-2 wrist epitope (residues 48–69) that can bind ALK-3 [241]. GBMP1a activates, although to a lesser extent than BMP-2, the Smad1/5/8 pathway in primary human brain tumor cells (glioblastoma). GBMP1a also limits the ability of glioma stem cells to self-renew, but favors their astroglial differentiation (Table 2) [241]. Even though the role played by astrocytes in neuroinflammation and human AD brain is still poorly understood, a peptide inducing astroglial differentiation may be of interest [278].

### 4.3. Peptides Derived from FGF and Other Factors

#### 4.3.1. FGF-2

- Peptide derived from FGF-2

Baird et al. have analyzed the functional domains in the primary sequence of FGF-2 responsible for heparin binding. Among 25 fragments, two interesting sequences have been identified: FGF (24-68) and FGF-2 (93-120). Both peptides can inhibit FGF-2 binding to its receptors on BHK cells. However, the shorter peptide FGF (106-115) is 10- to 100-fold more potent [279]. Furthermore, FK18, a peptide corresponding to the sequence FGF-2 (93-110), shows neuroprotective effects against excitotoxic injury [242] and has no toxic effect in vivo after its intravitreal administration. [280] (Table 2). The dimeric FGF2-FGFR1c structure has also revealed various FGF-2 β loop–strand regions acting as FGFR1 interaction sites [281]. This information was used to design different mimetic FGF-2 peptides (canofin1, canofin2, and canofin3) that were produced as tetrameric dendrimers coupled to a three-lysine backbone [282]. All three canofins bind to FGFR1 with a lower affinity than FGF-2. However, they induce neuronal differentiation, as shown by neurite outgrowth from rat cerebellar granule neurons, and protect differentiated neurons from apoptosis [282].

Because of their neuroprotective properties and neuronal differentiation effect, such peptides may be interesting molecules to fight degenerative disease.

#### 4.3.2. Other Growth Factors

- Peptides derived from IGF

A short tripeptide GPE derived from IGF-1 gave promising results as neuroprotective molecule both in vitro and in vivo using experimental models of neurodegeneration and brain trauma [283,284]. GPE is naturally released after the N-terminal cleavage of IGF-1 in the brain [285]. The synthetic GPE peptide increases the acetylcholine release in rat cortical brain slice in culture [286], and prevents the neuronal death in the hippocampus injured by NMDA in vitro [287]. It can cross the BBB upon its intraperitoneal administration, and reduces neuronal loss in the hippocampus after hypoxic–ischemic injury in adult rats [284].

To improve its pharmacokinetic, GPE was modified by adding an α-lipoic acid (LA-GPE, R-α-Lipoyl-GPE dimethyl ester). Both GPE and LA-GPE prevent the death of SH-SY5Y cells induced by Aβ_1–42_ in vitro. GPE-LA also reduces Aβ-induced AChE activity and oxidative stress [288]. To increase its stability in the blood and reduce its degradation by proteases, the amide bonds in GPE was replaced with an aminomethylene unit ψ[CH_2_NH] at Gly-Pro (GPE3), Pro-Glu (GPE1), or at both junctions (GPE2). As expected, GPE2 was more stable than GPE1 and GPE3, with half-lives of 11.8 h, 4.5 h, and 6.6 h, respectively. However, GPE3 had the best neuroprotective properties [289].

The GPE-modified peptides can block the effect of Aβ_1–42_, limiting inflammation and oxidative stress in vitro. They may therefore be considered for future application in neurodegenerative diseases such as AD.

- Peptides derived from CNTF

CNTF is well known for its neuroprotective effect [221]. Two peptides, P6 and P021, were derived from the biologically active region of human CNTF (amino acid residues 146–156) [290]. The peptide P6 is very stable over time, with a plasma half-life of over 6 h as compared to 3 min for CNTF. It can also cross the BBB [291]. Both P6 and P021 gave promising results in the context of AD in several mice models [292,293,294]. The intraperitoneal administration of P6 for six weeks in 6–7-month-old 3xTg-AD mice (prior to Aβ plaque and neurofibrillary tangle formation) limits the impairment in spatial memory [292,293]. It potentiates the neurogenesis in APP transgenic (Tg) mice by increasing cell proliferation [294]. In the same way, P021 enhances the proliferation and differentiation of adult hippocampal progenitors and improves cognition in C57Bl/6 mice and aged rats, favoring the synthesis of BDNF [295,296]. Therefore, these peptides derived from CNTF are also promising molecules to fight AD hallmarks.

In summary, the peptides derived from growth factors not only successfully mimic the receptor binding sites, but also initiate specific signaling pathways such as PI3K/AKT, MAPK/ERK1/2, and canonical Smad1/5/8 cascades involved in neuroprotective activity (GK-2, FK-18), neurogenesis (GK-2), and cholinergic differentiation (SpBMP-9, pBMP-9). Interestingly, some of them such as dNGF(1-15) and B-3/B-5 also promote the synthesis of BDNF and the expression of its receptors, while others (GK-2; P06 and P021) counteract the impaired cognitive functions in AD mice models without side effects. Furthermore, peptides like GK-2 did not induce hyperalgesia, which is one of the primary adverse effects of the native NGF protein. However, the side effects of peptides derived from growth factors such as pBMP-9 and SpBMP-9 are still poorly known and require further studies.

## 5. Conclusions

Finding a therapy for AD disease is one of the greatest challenges for modern medicine, since it is a multifactorial disease. Currently, major clinical trials are mainly focusing on Aβ hypothesis components, but have been largely unsuccessful. None of the available drugs protects against the loss of neurons, a hallmark in AD pathogenesis. In this regard, the exogenous administration of peptides derived from growth factors is an attractive therapeutic approach, given their roles in proliferation, differentiation, plasticity, and survival of neuronal cells. The strong supportive preclinical data in primary cells and animal models indicate the potential/viability of this strategy for AD treatment. Several short peptides derived from neurotrophins (NGF, BDNF), members of the TGF beta superfamily (BMP), and FGF have been developed or are under development to replace the deficient or dysregulated growth factor in AD. An advantage of these peptides is that their structure can be constrained (or designed) to better interact with the growth factor receptors and to activate a specific downstream pathway such as MAPK/ERK versus PI3K/AKT to favor a subsequent behavior like neuronal survival and/or differentiation. It also offers the opportunity to develop new therapeutic strategies by combining some of these peptides together or with other available treatment for a multimodal approach. Blood stability and pharmacokinetic properties of these peptides can also be improved by chemical modification. Their small size facilitates the penetration of the BBB to reach neuronal cells. However, systemic administration of these peptides could lead to serious peripheral side effects by acting on receptors in other tissues. Intranasal delivery or encapsulated cell biodelivery methods could help to overcome this limitation. Interestingly, if started early in the progression of the disease, this treatment could alter the relentless cognitive decline. However, future studies are required to better understand and improve the efficacy of these promising molecules in the context of AD pathogenesis.

## Figures and Tables

**Table 1:** Effect of the growth factor superfamily on CNS cells and their potential effect on Alzheimer’s disease hallmarks.

Superfamily	Experimental Conditions	Effect on CNS Cells In Vitro or In vivo	Refs
***Neurotrophin***			
**NGF**	***Animal:*** Homozygous *Ngf ^−/−^* knockout or WT mice C57BL/6	*Ngf* cKO mice:	[100]
		↓ Hippocampal Ngf mRNA level	
	***Treatment:*** Injection of viral vector for *Ngf* overexpression under stereotaxic guide	↓ Adult hippocampal neurogenesis	
		↓ Cholinergic fiber density in the hippocampus but not in the cortex	
		NGF restores hippocampal cholinergic fiber innervations and spatial memory.	
**BDNF**	***Animal:*** Female APP.PS1 transgenic mice	BDNF + ADTC5 compared to BDNF alone or vehicle:	[103]
		↑ Cognitive performance (Y-maze and new object recognition)	
	***Treatment:*** BDNF at 5.7 nmol/kg, BDNF + ADTC5 (modulator to allow BDNF to pass BBB) at 10 µmol/kg or vehicle. Intravenous injection every 4 days for 8 injections	↑ Degree of neuron-glial antigen 2 (NG2) receptor expression a marker for oligodendrocyte maturation	
		↑ Hippocampus level of early growth response 1 (EGR1) and activity-related cytoskeleton-associated protein mRNA transcripts	
		No significant impact on Aβ plaque	
	***Animal:*** AD11 anti-NGF mice (sporadic AD model), 6 months old	Rescue memory performance (object recognition and object context tests)	[104]
	***Treatment:*** Intranasal delivery at 12.6, 42, and 420 pmol/administration, repeated 7 times for 15 days	No impact on Ab plaques, tau hyperphosphorylation and cholinergic deficit	
		↓ CD11b-positive microglia in the hippocampus	
***BMP***			
**BMP-6**	***Cells*:** neuronal progenitor cells from adult rat (NPC)	Aβ_1-42_: ↑ BMP-6 level	[105]
	***Treatment:*** 50–100 ng/mL for 4 days with a refresh of medium containing BMP-6 at 2 days	BMP-6: ↓ Proliferation of NPC (dose dependent effect)	
**BMP-9**	***Animal*:** APP.PS1/CHGFP (AD model) and WT/CHGFP (control) transgenic mice 5 and 10 months old	↓ Number Aβ amyloid plaques in AD model	[106]
		↑ ChAT expression in APP.PS1/CHGFP and WT/CHGFP	
		↑ Density of cholinergic fibers in APP.PS1/CHGFP and WT/CHGFP	
	***Treatment*:** intraventricular infusion at 4 ng/h for 7 days	↑ Hippocampal level of receptors TrkA and p75NTR in 5 months old mice but not in 10 months old mice	
		↑ Hippocampal level of NGF in both mice (15–20%)	
		↑ IGF-1 levels in 5 months APP.PS1/CHGFP	
	***Animal*:** APP/PS1 mice (7 months)	Improve spatial and associative learning and memory (Morris water maze, contextual fear conditioning test)	[107]
		↓ Aβ levels and number of plaques in AD model	
	***Treatment:*** intranasal delivery of 50 ng/g/d for 30 days	↓ Hyperphosphorylated tau in the cortex and hippocampus	
		↓ Neuroinflammation (activated microglia and astrocytes)	
		↑ Expression of low-density lipoprotein receptor-related protein 1 (LRP1), involved in the clearance of Ab	
***IGF***			
**IGF-2**	***Animal:*** Tg2576 AD mouse (10 months old) and WT C57BL/6 mouse (control)	↓ Oxidative stress	[108]
		↑ Expression of *PI3K*, *AKT* and *CREB* in hippocampus ↓ levels of amyloid plaques in the hippocampus	
	***Treatment:*** Injection saline/DMSO (control) and IGF-2 at 250 ng	↑ Memory consolidation (Morris Water Maze) via PI3K/AKT pathway	
		↓ Memory decline	
	***Animal:*** APP.PS1/CHGFP and Wild Type (control) mice	↓ Aβ plaque numbers	[109]
		↑ p75NGFR compared to vehicle both in APP.PS1/CHGFP and WT	
		↑ ChAT in APP.PS1/CHGFP and WT hippocampus	
	***Treatment:*** Infusion of vehicle or 50 ng/h hIGF-2 for 7 days	↑ BMP-9 level in APP.PS1/CHGFP and WT hippocampus (basal level is higher in APP.PS1/CHGFP)	
		↓ ALK1 expression in WT but not in APP.PS1/CHGFP hippocampus	
		↓ FGF-2 level in APP.PS1/CHGFP hippocampus	
		↑ Hippocampal neurogenesis (DCX) in APP.PS1/CHGFP and WT hippocampus	
***FGF***			
**FGF-2**	***Animal:*** APP.PS1 mice (AD model) and WT Tg2576 mice (control).	**In vivo:** FGF-2 in APP.PS1 mice Reverse learning deficit memory ↓ Aβ hippocampal deposition ↑ Neurogenesis in subgranular zone of dental gyrus **In vitro:** FGF-2 ↑ Dose-dependent Aβ phagocytosis in microglia ↓ Production of Aβ in neural stem cells ↑ Neuronal differentiation of neural stem cells	[110]
	***Treatment:*** hippocampal injection of hybrid virus expressing FGF-2 or GFP (control) at 1 × 10^10^ per brain at 4 months (presymptomatic) and 7–8 months (postsymptomatic) of age		
	***Cells:*** Primary microglia culture and neural stem cells (mouse embryonic brain day 14)		
	***Treatment:*** Microglia: FGF-2 (0.1 or 1 ng/mL) + 10 µg fibrillar Aβ_1–42_ for 1 h		
	Neural stem cell: hybrid virus + 1 µM Aβ_1–42_ oligomer for 7 days		
	***Cells*:** Primary astrocyte culture weight FGF-2 (HMW, 23 kDa) at 10 ng/mL	LMW and HMW FGF-2:	[111]
		Induce ERK and AKT pathway activation	
		Protective effect against cytotoxicity induced by Aβ (20 μM) or oxidative stress	
	***Treatment:*** Medium with or without purified low molecular weight FGF-2 (LMW, 17 kDa) or high molecular	↑ Bcl-XL transcripts	
		LMW FGF-2:	
		↑ Proliferation by upregulation of c-Myc, Cyclin D1, and Cyclin E through PI3K/AKT pathway	
**FGF-21**	***Animal:*** male APP.PS1 transgenic mice (6 months old)	**In vivo:**	[112]
		Subcutaneous injection:	
		↑ Learning abilities after 5 days (Morris water maze)	
		↓ Brain Aβ burden	
	***Treatment*:** Subcutaneously injection with 5 mg/kg/day twice a day for 1 month or intracerebroventricular with a mini pump 0.4 µg/day for 14 days	↓ Tau phosphorylation positive area	
		Intracerebroventricular injection:	
		Rescue neurodegeneration through the FGF-21/FGFR1 signaling pathway (Morris water Maze)	
		**In vitro:**	
	***Cells:*** Rat (PC12) pheochromocytoma cells and rat astrocyte (C6) line in coculture treated with FGF-21 at different concentrations between 0.07 and 8 µM	↑ Cell viability against Aβ_25-35_ toxicity (higher effect in the presence of astrocytes)	
		↓ Tau hyperphosphorylation	
		↓ ROS levels	
		Rescues the lactate system deficiency induced by Aβ_25–35_	

**Table 2:** Effect of the peptides derived from growth factors on CNS cells and their potential effect on Alzheimer’s disease.

Superfamily	Peptide Sequence	Experimental Conditions	Effect on CNS Cells In Vitro or In Vivo	Refs
***Neurotrophin***				
**NGF**	**Dimeric dipeptide**:	***Cells*:** mouse hippocampal HT-22 immortalized neurons and primary culture of embryonic rat hippocampal neurons (18 days old embryos)	Strong neuroprotective properties at 10^−8^ M	[230]
	GK-2 β-turn loop L4	***Treatment*:** peptide (10^−5^–10^−10^ M) added 24 h before adding H_2_O_2_ (1.5 mM) for 30 min or glutamate (5 mM) for 24 h		
	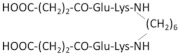	***Incubation time*:** 4 h and 24 h		
	**Dimeric dipeptides**	***Cells*:** mouse hippocampal HT-22 immortalized neurons and rat pheochromocytoma PC12	Both GK-2 (10^−8^ M) and GK-6 (10^−6^ M):	[231]
	GK-2 and		↑ Phosphorylation of TrkA	
	GK-6 β-turn loop L1	***Treatment*:** peptide (10^−5^–10^−10^ M) added 24 h before oxidative stress	GK-6 (10^−6^ M):	
	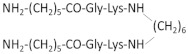		Exhibits slight neuroprotective properties	
			↑ Differentiation (↑ neurite outgrowth in PC-12 at 7 days)	
	**NGF N terminus**	***Cells*:** Rat PC12 pheochromocytoma cells	↑ Internalization of TrkA and p75NTR receptors	[232]
	**Linear peptide** NGF(1-14)			
	SSSHPIFHRGEFSV-NH_2_	***Treatment*:** peptide at 50 µM or NGF (50 ng/mL)	↑ Proliferation of PC12 cells at 48 h	
	**Dimeric peptide** d-NGF(1-15)			
	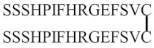	***Incubation time*:** 10 min to 72 h	↑ Differentiation (↑neurite total length at 72 h)	
	**Cyclic peptide**	***Cells*:** p75NTR- and TrkA-NIH-3T3 cells and E17 fetal rat cortical neurons	No effect on NGF (0.5 nM) binding to TrkA, supporting its specificity for p75NTR	[233]
			↓ Dose-dependent Aβ_1–40_ (0.5 nM) binding to p75NTR in rat cortical neurons	
	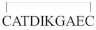	***Treatment*:** Cyclic peptide (0–300 nM) for 30 min or 24 h and then Aβ_1–40_ (0.5 nM, 25 nM or 20 µM) for 30 min	↓ Aβ_1–40_ (20 µM) signaling through p75NTR: ↓ c-jun mRNA and ↓ phosphorylation of cJUN	
			protects at 250 nM E17 neurons or 3T3 from Aβ_1–40_ (20 µM) -induced toxicity	
	**Dimeric dipeptide**:	***Animal*:** Mongrel male rats with bilaterally injection of Streptozotocin 3 mg/kg into their cerebral ventricles	GK-2 treatment can counteract the cognitive deficit in AD model (spatial memory impairment in Morris water maze)	[234]
	GK-2	***Treatment*:** GK-2 (0.5 mg/kg) or memantine (10 mg/kg) 4 h after the surgery and then once a day for 2 weeks	Effect similar to memantine	
	**Dimeric dipeptide**:	***Animal*:** Ischemic stroke animal model; male Wistar rats (8–9 weeks) with intravascular thread occlusion of the middle cerebral artery	↑ Hippocampal and striatum neurogenesis in rat cerebral ischemia	[235]
	GK-2	***Treatment*:** GK-2 (1 mg/kg, intraperitoneal); 6 or 24 h after surgery, once a day for 6 days	↓ Volume of the ischemic injury (60% when injected 6 h after surgery)	
	**Cyclic complex peptides derived from loops L1 and L4 with or without NGF N terminus**	***Cells:*** PC12 cells (clone 615) stably overexpressing TrkA, dorsal root ganglia (DRG) from 8-day-old chick embryos and cerebellar granule neurons from 8-day-old Sprague Dawley rat pups	**In vitro**:	[236]
			Both NL1L4 and L1L4 (3 µM) have neurotrophic properties	
	**NL1L4**	***Treatment***: NL1L4 (3, 6 and 10 µM), L1L4 (50, 100 nM, 3, 6 and 10 µM), and NGF (0.192 nM, control)	↑ DRG differentiation within 2 days like NGF	
	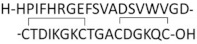		L1L4 dose-dependent ↑ PC12 differentiation at 3 days (EC_50_ 1 µM)	
		***Incubation time***: 10 min (TrkA activation); 2 weeks (DRG) and 3 days (PC12)	↑ TrkA phosphorylation (pTrkA) in PC12 cells at 10 min (NL1L4 and L1L4 (3 µM): 57 and 80% of pTrkA level obtained using NGF, respectively)	
			No effect on TrkB phosphorylation in cerebellar granule neurons	
	**L1L4**	***Animal***: CCI model (adult male Sprague Dawley rats) treated by L1L4 (37.5 µg/µL) through intrathecal lumbar spinal catheter (1 µL/h for 7 days)	**In vivo**:	
	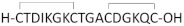		↓ Neuropathic pain in CCI model (restores mechanical and thermal sensitivity)	
**BDNF**	B-3 (Ac-SKKR-CONH_2_)	***Cells:*** NIH 3T3 cells transfected with TrkB receptor;	↑ TrkB phosphorylation at TrkB at Tyr 706 at 1 h	[237]
		mouse E18 primary	No cytotoxic effect on cells at 5 days	
		hippocampal neurons	↑ Neuronal differentiation (↑ b-III-tubulin, anti-neurofilament-M, and NeuN) in E18 hippocampal neurons at 5 days	
	B-5 (Ac-IKRG-CONH_2_)	***Treatment*:** peptides (2 nM to 10 µM)	↑ BDNF synthesis induced by B-3 (0.1 and 1 µM) and B-5 (0.1 µM) in primary E18 hippocampal cells at 5 days	
		***Incubation time*:** 1 h; 2 and 5 days	TrkB synthesis induced by B-3 and B-5 (1 µM) in NIH-3T3 at 5 days	
	GSB-106	***Animal*:** male C57Bl/6 mice (chronic social defeat stress (CSDS))	↑ Locomotion in CSDS mice	[238]
	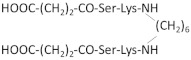	***Treatment*:** GSB-106 0.1 mg/kg once a day, for 21 days	Restores decreased synaptophysin level in hippocampus of CSDS mice	
***BMP***				
BMP-9	pBMP-9	***Cells*:** SH-SY5Y cells	↑ Neuronal differentiation (↑ neurite outgrowth; ↑ MAP-2, NSE. NeuN at 5 days).	[239]
	Ac-CGGKVGKACCVPTKLSPISVLYK-NH_2_	***Treatment*:** peptides or BMP-9 (control) at 0.1 or 1 nM with or without retinoic acid (RA) in serum-free medium	SpBMP-9 ↑ differentiation in cholinergic phenotype. (↑ acetylcholine, VAChT, ChAT) compared to BMP-9 or pBMP-9	
	SpBMP-9			
	Ac-CGGKVGKASSVPTKLSPISVLYK-NH_2_	***Incubation time*:** 1, 3, and 5 days.	Adding RA ↑ peptide-induced differentiation	
	SpBMP-9	***Cells*:** SH-SY5Y cells	SpBMP-9 plus NGF or bFGF	[240]
	and NSpBMP-9 (negative peptide)	***Treatment*:** peptides at 0.1 nM with or without NGF (100 ng/mL) or bFGF (FGF-2; 20 ng/mL) in serum-free medium	↑ Neuronal differentiation (↑ neurite outgrowth, ↑ NSE expression) compared to growth factor alone	
	Ac-CGGKVGKAGGVPTKLSPIGGLYK-NH_2_		↑ Neuronal differentiation in cholinergic phenotype. (↑ VAChT vesicles located in the neurites) compared with growth factor alone	
		***Incubation time*:** 5 days.	NSpBMP-9 has no effect	
BMP-2	GBMP1a (H-PFPLADHLNSTNHAIVQTLVNS-NH_2_)	***Cells*:** primary human glioblastoma cells (glioma stem cells subpopulation)	↑ Astroglial differentiation (↑ GFAP protein expression; ↑ S100)	[241]
		***Treatment*:** 60 ng/mL GBMP1a		
		***Incubation time*:** 5 days	↓ Cell proliferation	
***FGF***				
FGF-2	FK-18 FFFERLESNNYNTYSRK	***Cells*:** SH-SY5Y cell	↓ Glutamate-induced apoptosis via Akt activation	[242]
		***Treatment*:** FK18 at 10 µg/mL or bFGF at 100 ng/mL 2 h before, at the same time, or 30 min after stimulation with glutamate (4–10 mM)	↑ Bcl-2/Bax ratio	
			↓ Cleaved caspase-3	

## Data Availability

Not applicable.

## Abbreviations

AAV2-NGF	Adenoviral vector to deliver NGF
ActR-1A	Type 1A activin receptor
ActRIIA	Type II activin receptor
ActRIIB	Type IIB activin receptor
AD	Alzheimer’s disease
ADAM17	A disintegrin and metalloproteinase 17
ALK	Activin receptor-like kinases receptor
AMH/MIS	Anti-Müllerian hormone/Müllerian inhibiting substance
AMHRII	Anti-Mullerian hormone receptor type II
AMPA	Alpha-amino-3-hydroxy-5-methyl-4-isoxazolepropionate
APOE	Apolipoprotein E
APOE4	Apolipoprotein E4
APP	Amyloid-β precursor protein
AVVS2-NGF	Adeno-associated virus serotype 2 delivering NGF
Aβ	Amyloid-β
BAMBI	BMP and activin membrane-bound inhibitor
BBB	Blood–brain barrier
BDNF	Brain-derived neurotrophic factor (mature form: mBDNF)
BFCNs	Cholinergic neurons of the basal forebrain
BMP	Bone morphogenetic protein (mature form)
BMPR-1A	Type 1A BMP receptor
BMPR-1B	Type 1B BMP receptor
BMPRII	Type II BMP receptor
CAM	Calmodulin kinase
CDC42	Cell division control protein 42 homolog
ChAT	Acetylcholine synthesis enzyme
CNS	Central nervous system
CNTF	Ciliary neurotrophic factor
CR	Cysteine clusters
CREB	cAMP response element-binding protein
DAG	Diacylglycerol
DMSO	Dimethyl sulfoxide
dpp	Drosophila decapentaplegic
ERK1/2	Extracellular signal-regulated kinase
FDA	Food and Drug Administration
FGF	Fibroblast growth factor
FGFR	Fibroblast growth factor receptor
FRS2	Fibroblast growth factor receptor substrate 2
Gab1	Grb2-associated binder-1
GDF	Growth differentiation factors
GDNF	Glial derived neurotrophic factor
GFAP	Glial fibrillary acidic protein
GPCRs	G-protein-coupled receptors
Grb2	Growth factor receptor-bound protein 2
GSK3β	Glycogen synthase kinase-3-β
GWAS	Genome-wide association
HMW	High molecular weight
Ig-C2	Immunoglobulin-like C2 type domains
IGF	Insulin-like growth factor
IP3	Inositol 1,4,5-trisphosphate
I-Smad	Inhibitory Smad: Smad6/7
JNK	cJun N-terminal kinase
Lhx8	LIM homeobox 8
LMW	Low molecular weight
LOAD	Late-onset Alzheimer’s disease
LRR1-3	Leucine-rich 24-residue motifs
LTP	Long-term potentiation
MAGE	Melanoma-associated antigen
MAP-2	Microtubule associated protein 2
MAPK	Mitogen-activated protein kinase
MMP-9	Matric metalloprotease
NeuN	Neuronal nuclear protein
NF-kB	Nuclear factor kB
NG2	Neuron-glial antigen 2
NGF	Nerve growth factor (mature form: mNGF)
NMDA	N-methyl-D-aspartate
NMDAR	NMDA receptor
NRAGE	Neurotrophin receptor-interacting MAGE protein
NSE	Neuron specific enolase
NT-3	Neurotrophin-3
NT-4/5	Neurotrophin-4/5
p75NTR	p75 neurotrophin receptor
PI3K/AKT	Phosphoinositide 3-kinase/protein kinase B
PIP2	Phosphatidylinositol 4,5-bisphosphate
PKC	Protein kinase C
PLCγ	Phospholipase Cγ
PPM1A	Protein phosphatase magnesium-dependent 1A
RAF	Rapidly accelerated fibrosarcoma
RhoGDI1	RhoGDP dissociation inhibitor 1
Shc	Src homology 2 domain containing
Smad	Small mothers against decapentaplegic
SOS	Salt overly sensitive
STAT	Signal transducer of activators of transcription
TAB1/2/3	TAK1-binding protein 1/2/3
TAK1	Transforming growth factor β-activated kinase 1
TF	Transcription factor
Tg	Transgenic
TGF-β	Transforming growth factor-β
TRAF	TNF receptor associated factor
TREM2	Triggering receptor expressed on myeloid cells 2
Trk	Tropomyosin receptor kinase
Trk-FL	Trk full length
VAChT	Vericular acetylcholine transporter
WHO	World Health Organization
WT	Wild type
XIAP	X-linked inhibitor of apoptosis
